# Supplementary material for: Identification of photoperiod-induced specific miRNAs in the adrenal glands of Sunite sheep (Ovis aries)
Source: Front Vet Sci. 2022 Jul 22;9:888207. doi: 10.3389/fvets.2022.888207 (PMC9354845; doi:10.3389/fvets.2022.888207)
Supplement: Supplementary file 1 [file Data_Sheet_1.ZIP › Supplementary file/miRDeep2 file.zip!/miRDeep2 file/Know_Structure/SP42A1/oar-mir-410.pdf]

miRBase precursor : oar-mir-410
Total read count : 628
oar-miR-410-5p read count : 1
oar-miR-410-3p read count : 625
remaining reads : 2

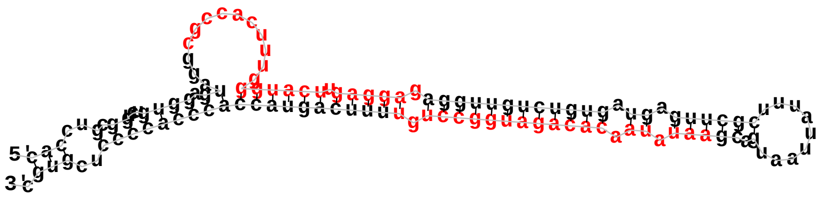

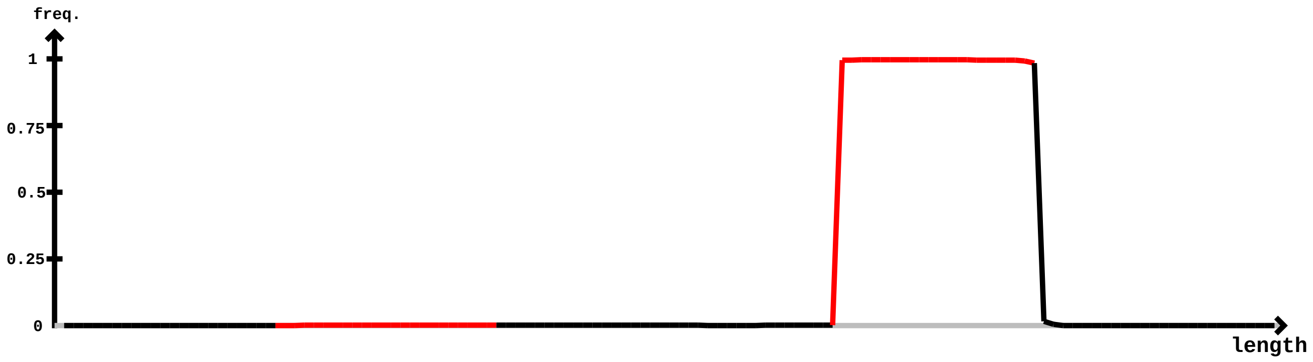


```
                                      oar-miR-410-5p
                                                                                                oar-miR-410-3p
5'- caccugcggucuguggaguaggcgccacuuuggguacuugaggagagguugucugugaugaguucgcuuuauuaaugacgaauauaacacagauggccuguuuucaguaccacccaccccucgugc   -3'    exp
    (((..(.(((..((((........))))....((((((.(((((.((((((((((((.((.((((((.........).))))).)).))))))))))))).)))))))))))....))).)..))).    reads       mm
    ........................ccacuuuggguacuugaggag.................................................................................    1           0
    .............................................agguugucugugaugaguucg............................................................    1           0
    .......................................................................uaaugacgaauauaacacagau.................................    1           0
    ................................................................................aauauaacacagauggccu...........................    2           0
    ................................................................................aauauaacacagauggccug..........................    5           0
    ................................................................................aauauaacacagauggccugu.........................    608         0
    ................................................................................aauauaacacagauggccuguu........................    7           0
    ................................................................................aauauaacacagauggccuguuu.......................    2           0
    ..................................................................................uauaacacagauggccuguuu.......................    1           0
```
